# DNA quantification of basidiomycetous fungi during storage of logging residues

**DOI:** 10.7717/peerj.887

**Published:** 2015-04-07

**Authors:** Isabella Børja, Gry Alfredsen, Tore Filbakk, Carl Gunnar Fossdal

**Affiliations:** Norwegian Forest and Landscape Institute, Ås, Norway

**Keywords:** Quantitative real-time PCR, Bioenergy, Forest slash, Fuel quality, Fungal colonisation

## Abstract

The demand for bioenergy caused an increased use of logging residues, branches and treetops that were previously left on the ground after harvesting. Residues are stored outdoors in piles and it is unclear to what extent fungi transform this material. Our objective was to quantify the amount of wood degrading fungi during storage using quantitative real-time PCR (qPCR) to detect basidiomycetous DNA in logging residues, a novel approach in this field. We found that the qPCR method was accurate in quantifying the fungal DNA during storage. As the moisture content of the piled logging residues decreased during the storage period, the fungal DNA content also decreased. Scots pine residues contained more fungal DNA than residues from Norway spruce. Loose piles had generally more fungal DNA than bundled ones.

## Introduction

Logging residues, also known as forest slash, are branches and tops left on the forest site after logging. In Norway spruce (*Picea abies* (L.) Karst.) and Scots pine (*Pinus sylvestris* L.) residues correspond to about 55 and 20% of the stem volume, respectively (Hakkila, 1991). In the past, the logging residues were uneconomical to gather and were left in the forest. With the increased need to utilize all renewable sources of energy, this lignocellulosic biomass provides a new potential as a fuel, e.g., chips for waterborne heating. Because loose logging residues are bulky to handle, they are sometimes compressed into bundles and piled for easier handling and transportation (Johansson et al., 2006). To promote drying of the material, piles are left in the forest or at the roadside for variable periods of time until further processing to final biofuel. While storage is used as a method to reduce moisture and thus makes the material better suited as fuel, the material also undergoes a transformation as it decomposes. Fungi are known as the main degraders of woody materials (Dix & Webster, 1995). However, to our knowledge the fungal colonization of unprocessed logging residues, tops and twigs, has not been quantified.

The moisture content and temperature are essential factors for fungal growth. Fungal decomposers are divided into three functional groups according to their substrate utilization pattern: white-, brown- and soft-rot fungi (Cooke & Rayner, 1984). White-rot fungi are mainly basidiomycetes and the only organisms known to be able to effectively utilize the lignin, cellulose and hemicellulose in various proportions (Cooke & Rayner, 1984). Brown-rot fungi, which appear to be exclusively basidiomycetes, utilize cellulose and hemicellulose, leaving the modified lignin in place (Cooke & Rayner, 1984). Soft rot decay by ascomycetes and mitotic fungi primarily occurs under conditions where the growth of the generally more active and competitive basidiomycetes is retarded (e.g., high moisture, low aeration). The decay caused by soft rot fungi is generally slower than decay caused by basidiomycetes. Basidiomycetes are likely the fungal group most responsible for the logging residue degradation (Tuomela et al., 2000).

Traditionally, to detect fungal biomass in soil and plant materials, the classical microscopic methods, and detection of specific cell wall components methods were used (Joergensen & Wichern, 2008). Also, the physiological method of selective respiratory inhibition, based on stimulation of respiration/metabolism of microorganisms by adding glucose to the substrate and subsequently inhibiting either fungi by adding cycloheximide or bacteria by adding streptomycin, was used (Anderson & Domsch, 1975). Flinkman & Thörnquist (1986) studied storage of bundled, unlimbed pulpwood and logging residues and found that the occurrence of microfungi in bundled material did not differ to any particular extent from loose material after eight months. They also noted that assortments with relatively large proportions of needles and bark appeared to provide the most favourable substrates for fungi. Jirjis & Lehtikangas (1993) studied fuel quality and dry matter loss during storage of logging residues in a pile. They found a general increase in spore count during approximately one year of storage and also an increase in viable spores. In another study by Lehtikangas & Jiris (1995) of logging residues in covered piles, the spore count at the end of the storage period (one year) was lower than at sampling after 7 months of storage, while the number of viable spores slightly increased. Spore counting does not discriminate between decay fungi and other fungal spores without additional isolation and identification in the lab. However, the development of DNA-based PCR (Polymerase Chain Reaction) and taxon-specific primers has provided a range of new possibilities. For example, Piskur et al. (2011) used PCR-DGGE method to analyze the fungal communities in degraded wood chips. Quantitative real-time PCR (qPCR) has proven to be a useful tool for the detection of plant pathogenic fungi and bacteria (Hietala et al., 2009; Salm & Geider, 2004; Schaad & Frederick, 2002; Schena, Hughes & Cooke, 2006; Vandroemme et al., 2008), estimation of fungal biomass in forest soil (Baldrian et al., 2013) but also wood deteriorating fungi (Eikenes et al., 2005; Pilgård et al., 2011; Pilgård, Alfredsen & Hietala, 2010; Song et al., 2014). The method is highly sensitive, specific and rapid, with the added capacity for quantification. To our knowledge, the qPCR approach has not so far been used to quantify the basidiomycetous fungi in logging residues.

The forestry practice of using the logging residues as a fuel chips is relatively recent and there is little available documentation on how microbial processes in stored piles may influence the final quality of the material as a fuel. Because the fungi degrade the wood and thus use up its energy, the amount of basidiomycetous DNA (indicative of the amount of fungal biomass) may be correlated with the degree of material degradation. Hence, we make the following assumption: the more basidiomycetous DNA measured, the higher the basidiomycetous colonization with degradation potential in the logging residue. By understanding the pattern of basidiomycetous colonization in stored logging residues better, this knowledge can be used to (1) better understand the potential effect of storage on basidiomycetous colonization and (2) optimize storage methods further.

This paper is based on the same samples as described in Filbakk et al. (2011). They modelled moisture content and dry matter loss during storage of logging residues. The first aim of this study was to implement quantitative qPCR as a novel technique to quantify the basidiomycete fungi in logging residues. The second aim was to find out how the storage conditions and type of forest residues influence the colonization by potentially decomposing basidiomycetous fungi.

## Experimental Section

### Experimental setup and sample taking

**Figure 1:**
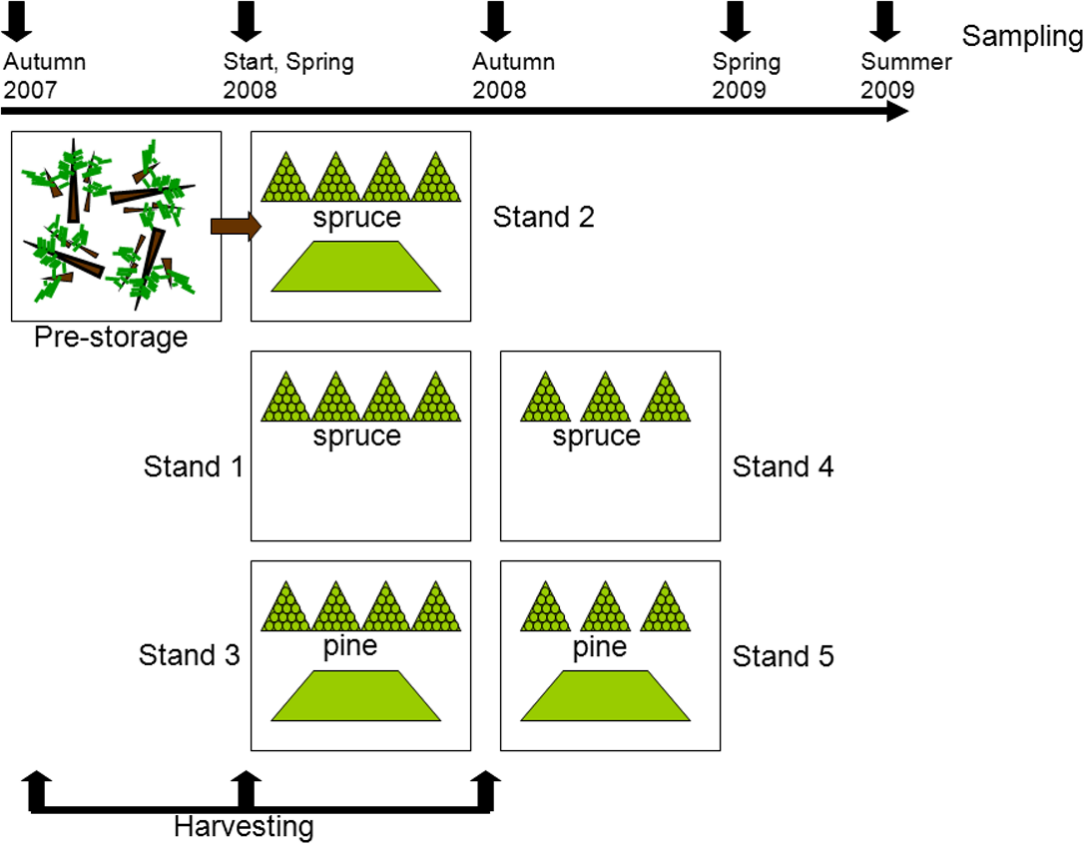
Experimental setup. After the first harvesting (felling of trees) the material was pre-stored by being left on the clear-cut during the winter. In the second and third harvesting periods piles of bundled (triangle symbol) and loose residues (trapezoid symbol) were constructed. At each sampling one bundled pile and a quarter of a loose pile were analyzed.

The detailed description of the experimental setup and sampling is given in Filbakk et al. (2011). Briefly, this study was carried out with residues originating from five different harvesting sites, all located close to Braskereidfoss (60°62′N/12°02′E), Norway. Three stands were predominantly Norway spruce (*Picea abies* (L.) Karst.) and two were dominated by Scots pine (*Pinus sylvestris* L.), all 70–100 years old. The experimental setup is illustrated in Fig. 1. Stand 2 was harvested in the autumn 2007 and logging residues were pre-stored; left lying on the clear-cut site until spring 2008 when piles were constructed. Stands 1 and 3 were harvested in spring 2008, and Stands 4 and 5 were harvested in autumn 2008. After each harvesting, the residues were stored in two types of piles, either in pyramid-like piles consisting of bundled residue material or loose piles consisting of unbundled material (Fig. 1). To protect the piles from precipitation, each pile was covered at the top with 2 mm thick residue wrapping paper produced by UPM Kymmene. At each sampling point the entire bundle was chipped in an industrial grinder (Peterson 4700B), resulting in about 1,000 kg of chipped material. From this source five replicates (1–2 kg each) were provided for further analysis. The chips were then removed before the next sample was chipped and sampled. Samples from piles were taken before storage (Start), then in spring 2008, autumn 2008, spring 2009, and summer 2009 (Fig. 1). All samples were analyzed for moisture content by the oven drying method at 103 °C (CEN/TS-14774-1, 2004), and for calorific value (CEN/TS-14918, 2005). To get a rough estimate of the dry matter loss in bundles, each bundle was weighed before placing into piles and at then before chipping by using a scale with accuracy of one kg. The total dry matter loss in bundled piles was calculated as the difference between initial and final mass of each bundle (Filbakk et al., 2011).

### DNA extraction

From each chipped sample, about 0.4 kg was randomly taken, dried at 50 °C for 24 h, and grinded first coarsely (Retsch Mühle grinder, 5 mm mesh; Retsch Gmbh, Haan, Germany) and then finely (IKA Werke MF 10 basic grinder, 0.5 mm mesh; IKA^®^-Werke Gmbh & Co., Staufen, Germany). The same sample batch was used for analyses by Filbakk et al. (2011). About 80 mg of finely grinded material was milled to powder-consistency in liquid nitrogen using a Retsch mixer mill (MM 300, Retsch Gmbh, Haan, Germany). Aliquots of 20 mg were prepared from powdered material for each treatment and total DNA was extracted using a DNeasy Plant Mini Kit (Qiagen, Hilden, Germany). The protocol provided by the manufacturer was followed. To account for the variation in DNA extractability in the environmental samples and to normalize for this variation, 5 ng of an external reference DNA, pGEM plasmid (pGEM-3Z Vector; Promega, Madison, Wisconsin, USA), was added to each sample upon start of each DNA extraction (Hietala et al., 2009). The extracted DNA was eluted in 50 µl of buffer AE and stored at −80 °C until processed by qPCR.

### Primer selection

The primers were based on prior findings by Vilgalys & Hester (1990) and Fierer et al. (2005) with minor modification to improve the number of basidiomycetes amplified. As a forward primer the 5.8sr TCGATGAAGAACGCAGCG primer was used (Fierer et al., 2005; Vilgalys & Hester, 1990) and as a reverse primer we selected a 2 nucleotide truncated ITS4-X primer CAG GAG ACT TGT ACA CGG TCC as it amplifies a larger set of basidiomycetous species. Primers for the internal pGEM control for extractability were selected as previously described by Coyne et al. (2005) and Pilgård, Alfredsen & Hietala (2010).

To test the primer specificity for basidiomycetes, pure cultures of organisms representing the target DNA (basidiomycetes) were used as positive controls (*Armillaria borealis* 2005-713/2, *Fomitopsis pinicola* 1946-755/2, *Coniophora puteana* 1982-97/3 and *Schizophyllum commune* 1956-1236/1), and as negative controls non-target DNA (aseptic spruce seedling roots, *Trichoderma* sp. 1959-1919/471, *Penicillium* sp. 2007-161/14/3 and *Cladosporium cladosporoides* 1967-149/11), all fungi obtained from the Norwegian Forest and Landscape Institute’s fungal culture collection (http://www.skogforsk.no/skogpatologi/database/searchform.cfm) were grown in petri dishes on cellophane over malt agar medium and incubated at 25 °C. To verify the specificity and sensitivity of the primers also on degraded wood samples, known dual mixtures of basidiomycetous DNA and host tree (*Serpula*-degraded and *Trametes*-degraded pine wood) together with internal control pGEM plasmid DNA were used. The presence of amplified qPCR product in the expected size range was verified by agarose gel runs. Negative controls consisted of both water and purified Scots pine DNA.

### qPCR conditions

The qPCR detection of basidiomycetous DNA (DNA_bas_) was performed using SYBR Green PCR Mastermix (Applied Biosystems, Foster City, California, USA) and the reference pGEM was quantified with TaqMan Universal PCR Master Mix (Applied Biosystems #4304437; Applied Biosystems, Foster City, California, USA).

The internal standard pGEM for calibrating DNA extractability was quantified as described by Coyne et al. (2005). The pGEM standard curve was prepared from serial diluted samples containing 0.066–0.000066 ng of pGEM DNA giving a standard curve of *y* = 2.7371 − 0.2642*x* (*x* is the Cq value and *y* the logarithmic amount of pGEM DNA present in the sample).

For quantification of DNA_bas_ a standard curve was prepared from DNA isolated from four basidiomycetous fungi (*A. borealis*, *F. pinicola*, *C. puteana* and *S. commune*), using serial diluted samples of 0.03–0.0003 ng of DNA giving a standard curve of *y* = 3.0922 − 0.176*x* (*x* is the Cq value obtained by qPCR and *y* the logarithmic amount of DNA_bas_ present in the sample).

For qPCR detection and quantification of DNA_bas_ the primer concentrations of 60 nM and 3 µl of extracted DNA solution from each sample were used for each reaction. The qPCR cycling was carried out using cycle parameters of 95 °C for 10 min to activate the polymerase, followed by 40 amplification cycles of 95 °C for 15 s and 60 °C for 1 min with signal thresholds set automatically.

The qPCR was performed with ABI PRISM 7700 (Applied Biosystems Foster City, California, USA). After amplification the data were analyzed and plotted (fluorescence vs. cycle number) using the Sequence Detection System, version 1.7a, Software Package (Applied Biosystems Foster City, California, USA). The extent of amplification was calculated as a mean Cq value of 2 technical replicates for each sample. All PCR reactions were performed in singleplex conditions under standard PCR cycling parameters. Undiluted, 10×, 100×, 1,000× and 10,000× diluted experimental sample concentrations were tested to investigate the presence of compounds inhibitory (impurities) to PCR amplification and to ensure that the amount of template fell in the linear range of the standard curve. The 100× dilution showed no indications of inhibition and will thus be presented. Five biological and two technical replicates were used for each sample.

### Statistical analysis

We analyzed the amounts of DNA_bas_ in logging residues from five forest stands as dependent parameter against the following independent categorical parameters: tree species, harvesting times, storage, within bundle placement, storage time. Precipitation, moisture, bundling time, mean temperature, dry matter loss and calorific value were continuous parameters in the model (Tables 1 and 2). The values for DNA_bas_ were normally distributed. To test whether the parameters had significant effects on the amount of DNA_bas_, we used analysis of variance (ANOVA) for all categorical parameters and linear regression for all continuous parameters. In addition, we tested interaction among wood moisture and storage method. First we tested whether pre-storage (leaving the logging residues on the harvesting site during the winter) had a significant effect on the DNA_bas_ between Stands 1 and 2. Because it did not, we included all five stands in the same model. Means in categorical parameters were compared by Tukey–Kramer HSD test at *P* < 0.05). In all cases, a null hypothesis was rejected at the 5% level of significance. All statistics were done using JMP (SAS Institute Inc., Cary, NC, USA).

**Table 1:** Description of parameters used in the study.

Parameter	Type	Explanation	Unit
Harvesting time	categorical	Season when the trees were cut (harvested)	Start, spring, autumn
Storage time	categorical	Days after the piles were made	Days
Precipitation	continuous	Mean precipitation since last sampling	Mm
Temperature	continuous	Mean temperature within piles	°C
Wood moisture	continuous	Moisture content measured in the sampled material	%
Tree species	categorical	Dominant tree species in the logging residue	Spruce, pine
Storage method	categorical	Piles made of loose or bundled forest residues	Start, loose, bundles
Calorific value	continuous	The amount of energy per kg given off when burnt	MJ/kg
Total dry matter loss	continuous	Total loss of dry matter during the storage, including fall off needles, twigs and microbial degradation	%
Placement	categorical	Location of bundles within piles	Top, bottom, middle
Pre-storage	categorical	Leaving the logging residue spread on the clear-cut site after harvesting, throughout the winter	Pre-storage, piles

**Table 2:** Test statistics and parameter estimates of the model.

	**Variance components:**	
*R* ^2^	0.68	
*R*^2^ adj	0.65	
RMSE (Root Mean Square error)	0.10	
N	133	
**Parameter estimates for the covariates in the model and *P*-values from effect tests:**		
	**Parameter estimate**	***P***-**values**
Intercept	0.3350	0.0024^*^
Storage time	−0.0004	<.0001^*^
Precipitation mm	0.0333	<.0001^*^
Storage method	–	<.0001^*^
Mean temperature	−0.0054	0.0040^*^
Harvesting time	–	0.0061^*^
Tree species	–	0.0091^*^
Wood moisture*storage method	–	0.0397^*^
Wood moisture	−0.0045	0.0398^*^
**Parameter estimates for categorical variables and Tukey–Kramer (T–K) 0.05 tests on LS means:**		
	**Parameter estimate**	**T–K**
Harvesting time [Autumn 2007]	0.0301	A
Harvesting time [Spring 2008]	0.0218	A
Harvesting time [Autumn 2008]	−0.0519	B
Tree species [pine]	0.0351	A
Tree species [spruce]	−0.0351	B
Storage method [Start]	−0.1393	C
Storage method [Bundle]	0.0334	B
Storage method [Loose]	0.1059	A
Wood moisture*storage method [Start]	0.0079	–
Wood moisture*storage method [Bundle]	−0.0026	–
Wood moisture*storage method [Loose]	−0.0053	–

**Notes.**

* Significance level 0.05.

## Results and Discussion

### qPCR assay

The qPCR primers used in our assay were specific for tested DNA_bas_, while the non-target DNA used as negative controls (water, host tree DNA, bacterial and non-basidiomycetous fungi) was not amplified under the conditions used. Likewise, the primers used for the calibration of DNA extractability between samples amplified only the pGEM plasmid used as an internal control in these studies. The qPCR can detect down to one average size fungal genome (i.e., one fungal cell) corresponding to the lowest point on our standard curve (∼0.0003 ng).

**Figure 2:**
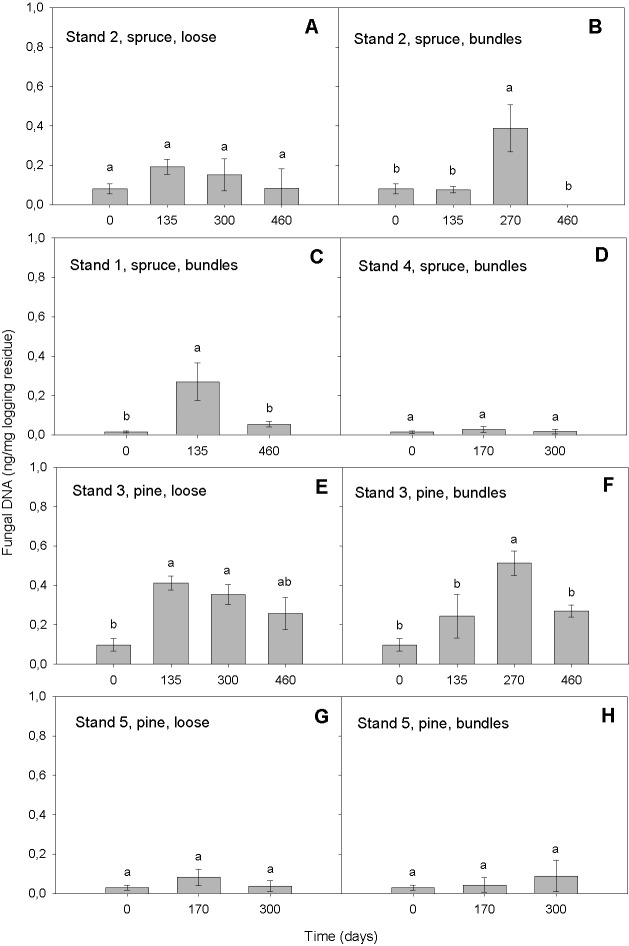
Amount of DNA_bas_ (ng/mg logging residue) detected in logging residue after increasing storage time (days) in all five stands, in loose and bundled piles of spruce and pine (*n* = 133). Standard errors are marked as vertical lines and means are compared by Tukey–Kramer test at *P* < 0.05, where different letters denote significant differences.

Our qPCR assay was able to quantify DNA_bas_ in logging residues. It detected DNA_bas_ in the range of 0.01–0.69 ng DNA_bas_/mg in chipped logging residue (Fig. 2). That the most elevated amounts of DNA_bas_ detected was after the highest precipitation event i.e., conditions promoting basidiomycetous growth (see below, ‘Variation in DNA_bas_ over time’ and ‘Moisture’) is an indicator that the sampling was representative and that the selected primers were efficient. This same general pattern was found in all stands: the lowest DNA_bas_ value was detected at the start of the storage period, followed by increase in the wetter period in spring 2009, followed by a decrease during the dryer period at the end of the storage, suggesting that our detected values may reflect the environmental conditions impact on basidiomycetous growth. Our DNA_bas_ values were considerably higher than those found by Pilgård et al. (2011) in Scots pine heartwood, preservative treated and furfurylated stakes in a soil contact (EN 252, 1989), when using qPCR. However, the substrates and exposure situation in these two studies were very different; Scots pine heartwood has some natural resistance to fungal decay and preservative treated or modified wood are commercial treatments used to hinder the basidiomycetous deterioration. In this study, the forest residues were not treated with wood protection systems in order to prolong their service life and thus were likely exposed to higher colonization potential by fungi, which may explain higher DNA_bas_ in samples tested in our study.

Although the qPCR assays are highly sensitive with high resolution and correlation to mass loss at the early stages of decay, they may lack accuracy in advanced decay stages due to substrate depletion (Eikenes et al., 2005). We did not have substrate depletion in our material, since the level of decay was at all times only in its initial stages of colonization and thus substrate availability was plentiful for further basidiomycetous growth when the environmental conditions were favorable.

The accuracy of the qPCR based quantification of fungal biomass depends on extraction efficiency and purity of extracted DNA. The mean and modus concentrations of extracted DNA in our samples were even 95 and 83 ng/µl, respectively. The ratio of absorbance at 260 nm and 280 nm is used to assess the purity of DNA. The mean and modus value for 260/280 ratio in our extracted DNA measured by NanoDrop (NanoDrop Technologies, Inc, Wilmington, Delaware, USA) was 1.8, which is accepted as “good quality” DNA. The absorbance ratio 260/230 is used as a secondary measure of nucleic acid purity and expected values are commonly in range 2.0–2.2. Mean and modus values in our samples were 1.4, suggesting that contaminants absorbing at 230 nm, such as carbohydrates or phenols, may have been present. Impact of contaminants was avoided by diluting samples, and dilution 100X ensured optimal accuracy.

In spite of the increasing use of qPCR for fungal biomass estimation, it is known that there is variation in ITS copy numbers (per ng DNA) among fungal species (Baldrian et al., 2013; Song et al., 2014), which will impact on the biomass estimation. We used a DNA from four different basidiomycetes to make our standard curves to partly accommodate for such ITS differences, but the obtained values must be considered as estimates of DNA_bas_. When Baldrian et al. (2013) compared fungal biomass content in litter and soil by using three different methods (ergosterol, phospholipid fatty acid and qPCR), they demonstrated that the obtained estimates led to large differences in relative amount of litter and soil fungal biomass. They estimated 28, 7 and 2 times larger fungal biomass in litter than soil by ergosterol, phospholipid fatty acid and qPCR, respectively, indicating that qPCR was the one least likely to overestimate the fungal biomass in litter.

### DNA_bas_ and storage conditions

The statistical model we used included data from all stands and explained 68% of the variability in the data (Table 2, *n* = 133, *R*^2^ = 0, 68). Of all the parameters we tested, only harvesting time, storage time, precipitation, temperature, wood moisture content, tree species and storage method significantly affected the amount of DNA_bas_ detected in the logging residues (Table 2). In addition, the wood moisture*storage method contributed significantly. As fungi colonize the material, fungal biomass, species diversity and material decomposition change. Therefore in our study, each time the sample was taken, offers a mere snapshot of the situation at the given time.

#### Variation in DNA_bas_ over time

**Figure 3:**
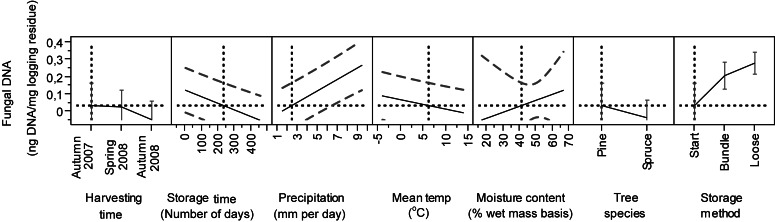
Predicted estimates of DNA_bas_ in logging residues (ng DNA/mg logging residue) in relation to harvesting time, storage time, precipitation, temperature, moisture content, tree species and storage method. Vertical and horizontal dotted lines show the current values for variables on *x* and *y*-axes. The black lines show how the predicted value of the variables on the *y*-axes change with changing of the values on the *x*-axes. The 95% confidence interval for the predicted values is shown by striped line surrounding the prediction trace (or error bar for categorical variables harvesting time, tree species and storage method).

In the model, the storage time affected the DNA_bas_ amount significantly (*p* < 0.0001). Generally, the amount of DNA_bas_ decreased at the end of the storage (Figs. 2 and 3). In all stands and pile types we detected the lowest DNA_bas_ values at the start, slightly rising during the storage and decreasing at the end of the study (Fig. 2). The transient DNA_bas_ increase was significant for the combinations illustrated in Figs. 2B, 2C, 2E and 2F.

The highest DNA_bas_ content was detected in bundles after 270 days, in spring 2009, in a Norway spruce stand (*n* = 5, mean 0.39 ng DNA, Fig. 2B) and in a Scots pine stand (*n* = 5, mean 0.51 ng DNA, Fig. 2F). Both peaks coincided with the highest precipitation (9 mm) measured during the entire study period, almost 5-fold higher than the mean precipitation.

The general decline in amount of DNA_bas_ with time may be due to transpiration drying, as transpiration continues from foliage or open wood surfaces after harvesting (Andersson et al., 2002). Indeed, such a decline in moisture has already previously been documented in this material by Filbakk et al. (2011).

Material from Stands 4 and 5, with the lowest DNA_bas_ values, was harvested and piled in autumn 2008 and had the shortest storage period. It is likely that the conditions were not conducive for fungal development during most of the storage period (winter/spring), since basidiomycetous fungi have restricted growth at low temperatures.

#### Seasonal effects

The model showed a significant contribution of the harvesting time (time of felling), to the amount of DNA_bas_ (Table 2). We detected significantly lower start DNA_bas_ values from material harvested in autumn 2008 than in autumn 2007 and spring 2008 (Table 2 and Fig. 3). The moisture content in the stored biomass tends to increase in late autumn and winter in the Nordic climate (Nurmi, 1999; Pettersson & Nordfjell, 2007). Although studies by Nurmi & Hillebrand (2007) showed that harvesting in spring gave better conditions for drying, thus less favorable conditions for fungi, and is in line with our observations.

#### Moisture

In the model, wood moisture (*p* = 0.0398), precipitation (*p* < 0.001) and the wood moisture*storage method (*p* = 0.0397) contributed significantly in explaining DNA_bas_ content (Table 2). The prediction profiles illustrate that DNA_bas_ increased with precipitation and wood moisture content. We found the highest values of DNA_bas_ in material with moisture 30–35% (Fig. 3). In general, as expected, the moisture content decreased during storage for all materials, in Stand 2 from 55 to 50%, Stands 1 and 3 from 45% to 30%, Stand 4 from 45 to 40% and Stand 5 from 55 to 40% (Filbakk et al., 2011). The average moisture content reduction was 12% larger in summer than in winter, the relative air humidity in the piles was about 100 % and the temperature in the piles closely followed the ambient temperature, due to fairly small piles, never exceeding 19 °C (Filbakk et al., 2011). The significant interaction of the moisture*storage method may be because the “Start” was included in our study as one of the storage methods (Table 2 and Fig. 3) and the moisture content was highest at this point.

Our results indicate that the moisture content in the piles was sufficient for growth of basidiomycetous fungi, never below 20%. A wood moisture content of 20% is often used as a threshold value for risk of fungal growth (Zabel & Morrell, 1992). Water is needed for fungal decay of wood because it (1) participates at hydrolysis, (2) serves as a diffusion medium, (3) is a solvent or life-supporting medium, (4) a mean for capillary swelling of wood (Zabel & Morrell, 1992). However, fungi can survive in a dormant state and rewetting will revive them (Findlay, 1950). In general, optimal moisture for growth of the majority of wood decaying basidiomycetes is in the range between 40 and 80% (Eaton & Hale, 1993).

Indeed, the high amount of precipitation (9 mm) in spring 2009, after 270 days of storage, was associated with higher DNA_bas_ values (Figs. 2B and 2F). The temporary peak DNA_bas_ values related to peak moisture in the material (Filbakk et al., 2011) further indicate that even a short period of high precipitation may increase the basidiomycetous growth.

#### Tree species

Tree species contributed significantly (*p* = 0.0091) to the amount of DNA_bas_ in logging residues (Table 2). The Tukey–Kramer test showed a significant difference between Scots pine and Norway spruce (Table 2). We found significantly more DNA_bas_ in pine-dominated residues (*n* = 64, mean 0.19 ng DNA_bas_) than in spruce-dominated ones (*n* = 69, mean 0.10 ng DNA_bas_), and there was larger variation in values in pine than in spruce residues (Figs. 2 and 3).

The significant differences between the tree species were mainly due to the high DNA_bas_ values we detected in pine stand 3. Pine stand 5 had very low values. Because the spruce has a higher percentage of small branches with needles the bundles are usually denser, with more moisture and less airflow, than pine bundles. The environment in pine bundles may have been more conducive to basidiomycetous growth than in spruce bundles.

The high variability among the DNA_bas_ found at the five different stands, with high values in pine stand 3 (mean 0.5 ng DNA_bas_) and correspondingly low values at spruce stand 4 (mean 0.02 ng DNA_bas_), probably contributed to the significant differences among the tree species.

#### Storage method

The storage methods alone (*p* < 0.0001) and also in combination with moisture (*p* = 0.0397) contributed significantly in explaining the amount of DNA_bas_ in logging residues (Table 2). We found significant differences in DNA_bas_ among the three storage methods. In all stands the amount of DNA_bas_ was, as expected, always lowest at start, before bundling or loose pile construction (*n* = 20, mean 0.04 ng DNA_bas_). Compared to start, the DNA_bas_ was significantly higher in bundles (*n* = 65, mean 0.16 ng DNA_bas_) and highest in loose residues (*n* = 48, mean 18 ng DNA_bas_).

Only minor differences were detected in the moisture content between bundles and loose piles (Filbakk et al., 2011). Hence the consistence of loose piles may have created microclimate with more air flow, thus more conducive to basidiomycetous development than in the compacted bundles.

We did not find any significant differences in DNA_bas_ amount related to material location within the pile. Filbakk et al. (2011) found the highest material moisture in the middle of the pile; however, the moisture content in this study was always within the favorable range for basidiomycetous growth (Eaton & Hale, 1993).

When comparing bundled spruce material, with or without pre-storing in Stands 1 and 2, we found that the mean DNA_bas_ content was similar, 0.11 and 0.13 ng DNA_bas_ respectively (*n* = 42, Figs. 2B and 2C). This may imply that the pre-storage on the harvesting site during the winter, before piling, does not increase the decay of the logging residues.

### Practical implications

Both drying (including fall off needles and twigs) and microbial activity will result in dry matter loss in logging residues. In Filbakk et al. (2011) about 20% total dry matter loss was measured for bundled residues. However, this dry matter loss was most probably caused by handling together with the process of transpiration drying, causing the dry foliage and small twigs to fall off. Because the error in exact measuring of the dry matter loss in bundles was large, the mass loss caused by fungi alone could not be specified. The needle and twig fall off is considered beneficial because (1) its presence lowers the final combustion efficiency and (2) the nutrients contained in this material remain at the site as opposed to nutrient depletion by its complete removal.

We show that moisture is a key factor for basidiomycetous growth in piles. During storage time of maximum 460 days the moisture content in logging residues gradually decreased together with DNA_bas_ content. Hence, our results indicate that that storage of logging residues for maximum 460 days under these conditions give low levels of decay. Because we found higher values of DNA_bas_ in loose residues compared to bundles, the practice of bundling does not seem to promote more basidiomycetous growth than leaving the residues in loose piles. Neither did pre-storing of residues during winter seem to make a difference to DNA_bas_ content.

Our data suggest that basidiomycetous growth may be temporarily stimulated when the material has the optimal water content, such as in periods of increased precipitation, This supports the practice of covering the piles during storage.

## Conclusions

• We show that qPCR assay to quantify the DNA_bas_ is a method, capable of detecting the basidiomycetous biomass in logging residue material.
• Because the DNA_bas_, indicating the presence of potentially decomposing basidiomycetes, decreases at the end of the storage period together with moisture content, there seems to be a small danger of fungi significantly transforming logging residues stored for less than 460 days at given conditions.
• Scots pine residues had more DNA_bas_ than Norway spruce residues.
• Loose piles had generally more DNA_bas_ than bundled ones.

## Supplemental Information

10.7717/peerj.887/supp-1 Supplemental Information 1 Background data

## References

[ref-1] Anderson JPE, Domsch KH (1975). Measurement of bacterial and fungal contributions to respiration of selected agricultural and forest soils. Canadian Journal of Microbiology.

[ref-2] Andersson G, Asikainen A, Björheden R, Hall PW, Hudson JB, Jirjis R, Mead DJ, Nurmi J, Weetman GF, Richardson J, Björheden R, Hakkila P, Lowe AT, Smith CT (2002). Production of Forest Energy. Bioenergy from sustainable forestry: guiding principles and practice.

[ref-3] Baldrian P, Vetrovsky T, Cajthaml T, Dobiasova P, Petrankova M, Snajdr J, Eichlerova I (2013). Estimation of fungal biomass in forest litter and soil. Fungal Ecology.

[ref-4] CEN/TS-14774-1 (2004). Solid biofuels- methods for determination of moisture content- oven dry method, part 1: total moisture- reference method.

[ref-5] CEN/TS-14918 (2005). Solid biofuels- method for determination of calorific value.

[ref-6] Cooke RC, Rayner ADM (1984). Ecology of saprotrophic Fungi.

[ref-7] Coyne KJ, Handy SM, Demir E, Whereat EB, Hutchins DA, Portune KJ, Doblin MA, Cary SC (2005). Improved quantitative real-time PCR assays for enumeration of harmful algal species in field samples using an exogenous DNA reference standard. Limnology and Oceanography-Methods.

[ref-8] Dix NJ, Webster J (1995). Fungal ecology.

[ref-9] Eaton RA, Hale MDC (1993). Wood: decay, pests, and protection.

[ref-10] Eikenes M, Hietala AM, Alfredsen G, Fossdal CG, Solheim H (2005). Comparison of quantitative real-time PCR, chitin and ergosterol assays for monitoring colonization of *Trametes versicolor* in birch wood. Holzforschung.

[ref-11] Fierer N, Jackson JA, Vilgalys R, Jackson RB (2005). Assessment of soil microbial community structure by use of taxon-specific quantitative PCR assays. Applied and Environmental Microbiology.

[ref-12] Filbakk T, Høibø OA, Dibdiakova J, Nurmi J (2011). Modelling moisture content and dry matter loss during storage of logging residues for energy. Scandinavian Journal of Forest Research.

[ref-13] Findlay WPK (1950). The resistance of wood-rotting to desiccation. Forestry.

[ref-14] Flinkman M, Thörnquist T (1986). Lagring av buntade träddelar och hyggesrester.

[ref-15] Hakkila P (1991). Hakkuupoistauman latvusmassa. Folia Forestalia.

[ref-16] Hietala AM, Nagy NE, Steffenrem A, Kvaalen H, Fossdal CG, Solheim H (2009). Spatial patterns in hyphal growth and substrate exploitation within Norway spruce stems colonized by the pathogenic white-rot fungus *Heterobasidion parviporum*. Applied and Environmental Microbiology.

[ref-17] Jirjis R, Lehtikangas P (1993). Bränslekvalitet och substansförluster vid vältlagring av hyggesrester (Fuel quality and dry matter loss during storage of logging residues in a windrow). Report nr 236.

[ref-18] Joergensen RG, Wichern F (2008). Quantitative assessment of the fungal contribution to microbial tissue in soil. Soil Biology & Biochemistry.

[ref-19] Johansson J, Liss J-E, Gullberg T, Björheden R (2006). Transport and handling of forest energy bundles—advantages and problems. Biomass and Bioenergy.

[ref-20] Lehtikangas P, Jiris R (1995). Hyggesrester i täckta vältor—Nedbördens inverkan på bränslekvaliteten (Logging residues in covered windrows—Influence of precipitation on fuel quality). Rapport nr 173.

[ref-21] Nurmi J (1999). The storage of logging residue for fuel. Biomass and Bioenergy.

[ref-22] Nurmi J, Hillebrand K (2007). The characteristics of whole-tree fuel stocks from silvicultural cleanings and thinnings. Biomass and Bioenergy.

[ref-23] Pettersson M, Nordfjell T (2007). Fuel quality changes during seasonal storage of compacted logging residues and young trees. Biomass and Bioenergy.

[ref-24] Pilgård A, Alfredsen G, Bjordal CG, Fossdal CG, Børja I (2011). qPCR as a tool to study basidiomycete colonization in wooden field stakes. Holzforschung.

[ref-25] Pilgård A, Alfredsen G, Hietala A (2010). Quantification of fungal colonization in modified wood: quantitative real-time PCR as a tool for studies on *Trametes versicolor*. Holzforschung.

[ref-26] Piskur B, Bajc M, Robek R, Humar M, Sinjur I, Kadunc A, Oven P, Rep G, Petkovsek SA, Kraigher H, Jurc D, Pohleven F (2011). Influence of *Pleurotus ostreatu*s inoculation on wood degradation and fungal colonization. Bioresource Technology.

[ref-27] Salm H, Geider K (2004). Real-time PCR for detection and quantification of *Erwinia amylovora*, the causal agent of fireblight. Plant Pathology.

[ref-28] Schaad NW, Frederick RD (2002). Real-time PCR and its application for rapid plant disease diagnostics. Canadian Journal of Plant Pathology-Revue Canadienne De Phytopathologie.

[ref-29] Schena L, Hughes KJD, Cooke DEL (2006). Detection and quantification of *Phytophthora ramorum*, *P. kernoviae*, *P. citricola* and *P. quercina* in symptomatic leaves by multiplex real-time PCR. Molecular Plant Pathology.

[ref-30] Song Z, Vail A, Sadowsky MJ, Schilling JS (2014). Quantitative PCR for measuring biomass of decomposer fungi in planta. Fungal Ecology.

[ref-31] Tuomela M, Vikman M, Hatakka A, Itävaara M (2000). Biodegradation of lignin in a compost environment: a review. Bioresour Technol.

[ref-32] Vandroemme J, Baeyen S, Van Vaerenbergh J, De Vos P, Maes M (2008). Sensitive real-time PCR detection of *Xanthomonas fragariae* in strawberry plants. Plant Pathology.

[ref-33] Vilgalys R, Hester M (1990). Rapid genetic identification and mapping of enzymatically amplified ribosomal DNA from several *Cryptococcus* species. Journal of Bacteriology.

[ref-34] Zabel RA, Morrell JJ (1992). Wood microbiology—decay and its prevention.

